# Individual metabolomic signatures of circadian misalignment during simulated night shifts in humans

**DOI:** 10.1371/journal.pbio.3000303

**Published:** 2019-06-18

**Authors:** Laura Kervezee, Nicolas Cermakian, Diane B. Boivin

**Affiliations:** 1 Centre for Study and Treatment of Circadian Rhythms, Douglas Mental Health University Institute, Department of Psychiatry, McGill University, Montreal, Canada; 2 Laboratory of Molecular Chronobiology, Douglas Mental Health University Institute, Department of Psychiatry, McGill University, Montreal, Canada; Fundacion Instituto Leloir, ARGENTINA

## Abstract

Misalignment of the daily sleep-wake and fasting-feeding cycles with the endogenous circadian timing system is an inevitable consequence of night shift work and is associated with adverse metabolic health effects. However, a detailed characterisation of the effects of night shifts on 24-h rhythms in the metabolome is missing. We performed targeted metabolomic profiling on plasma samples collected every 2 h from healthy human subjects during two 24-h measurement periods at baseline and on the fourth day of a simulated night shift protocol, in which the habitual sleep-wake cycle was delayed by 10 h. Thirty-two out of the 130 detected metabolites showed a 24-h rhythm both at baseline and during the night shift condition. Among these, 75% were driven by sleep-wake and fasting-feeding cycles rather than by the endogenous circadian clock, showing an average phase delay of 8.8 h during the night shift condition. Hence, the majority of rhythmic metabolites were misaligned relative to the endogenous circadian system during the night shift condition. This could be a key mechanism involved in the increased prevalence of adverse metabolic health effects observed in shift workers. On the individual level, the response to the night shift protocol was highly diverse, with phase shifts of rhythmic metabolite profiles ranging from a 0.2-h advance in one subject to a 12-h delay in another subject, revealing an individual metabolomic signature of circadian misalignment. Our findings provide insight into the overall and individual responses of the metabolome to circadian misalignment associated with night schedules and may thereby contribute to the development of individually tailored strategies to minimise the metabolic impacts of shift work.

## Abstract 2

A study of simulated night shifts in humans shows that 24-hour rhythms in plasma metabolites are primarily influenced by behavioural cycles rather than the endogenous clock; in addition, a large degree of variability between individuals suggests the existence of individual metabolomic signatures of circadian misalignment.

## Introduction

Around 20% of the workforce in industrialised countries is engaged in shift work [1–3]. Shift work is associated with an increased risk of developing various metabolic disorders, including diabetes, obesity, and cardiovascular disease [4]. Circadian misalignment, which occurs when the timing of sleep and food intake is shifted with respect to its conventional phase position relative to the endogenous circadian timing system, is thought to contribute to these shift work–related health issues [5]. Indeed, laboratory studies have shown that circadian misalignment leads to a range of adverse metabolic changes within a few days, such as reduced energy expenditure, insulin sensitivity, glucose tolerance, and decreased secretion of the appetite-suppressing hormone leptin [6–9]. Furthermore, a recent study in shift working female nurses observed changes across the metabolome in response to night shift work, most notably in early chronotypes [10].

The circadian clock is thought of as an adaptive system that enables organisms to optimally synchronise internal physiological and behavioural processes with predictable daily environmental fluctuations in light, temperature, and food availability [11]. The circadian system consists of a central clock located in the suprachiasmatic nuclei (SCN) of the hypothalamus and peripheral clocks that are found in nearly every cell type in the body [12]. Driven by transcriptional-translational feedback loops involving a set of core clock genes that regulate the transcription of numerous clock-controlled genes in both the SCN and in peripheral tissues, the circadian system exerts a profound influence on physiology [13]. As a result, many metabolic processes show circadian rhythms that, under normal physiological circumstances, are synchronised with behavioural cycles of sleeping-waking and fasting-feeding.

Various metabolomic studies in humans have revealed extensive circadian and diurnal variation across diverse metabolite classes [14]. For example, the levels of 15% of metabolites were found to display a circadian rhythm independent of sleep or food intake in an unbiased screen of the plasma metabolome in healthy human subjects [15]. In experimental studies in which sleep-wake and fasting-feeding cycles are conventionally aligned with the endogenous circadian clock, a higher number of metabolites show diurnal variations in their levels, with estimates ranging from 19% to 64% [16,17], indicating the strong effect of behavioural cycles on oscillating metabolite levels. Together, these studies have advanced the understanding of the complex interplay between circadian and behavioural influences on circulating metabolite levels [18]. However, little is known about the effect of circadian misalignment on the human metabolome. It was previously shown in healthy human subjects in whom the sleep period was shifted to the daytime that daily rhythms in the levels of plasma metabolites are predominantly driven by behavioural (e.g., sleep-wake and fasting-feeding) cycles, creating a state of desynchrony between the central circadian clock and metabolic processes in peripheral tissues [19]. Currently, a detailed characterisation of the effect of night shifts on the human circadian metabolome and the interindividual variability in this effect is missing.

The aim of this within-subject laboratory study was to determine the impact of a 4-d simulated night shift protocol, in which habitual sleep periods are delayed by 10 h, on 24-h profiles of plasma metabolites in healthy human subjects. We also sought to determine individual differences in the metabolomic response to the shifted behavioural cycles. While on the group level we found that metabolite rhythms were primarily influenced by the shifted behavioural cycles, our results indicate that individual responses to the night shift condition were extremely diverse: in some subjects, metabolite rhythms largely shifted along with the 10-h delay in behavioural cycles, whereas in other subjects there was no evidence of adaptation.

## Results

Nine healthy human subjects (8 men, 1 woman, naturally ovulating and studied during her follicular phase; aged between 18 and 29 [mean: 23] years; BMI between 19.6 and 23 [mean: 21.3] kg/m^2^) completed a 4-d simulated night shift protocol in which the usual sleep period was delayed by 10 h (S1 Fig). Plasma samples were collected every 2 h during two 24-h measurement periods at baseline and on the fourth day of the night shift protocol. During the wake episodes of the measurement periods, subjects consumed hourly isocaloric snacks. A total of 229 samples were available for metabolomic profiling. Using quantitative targeted metabolomic profiling, 132 metabolites were detected in our samples. Concentrations of 2 detected metabolites (3-hydroxyphenyl)-3-hydroxypropionic acid [HPHPA] and putrescine) were below the limit of detection (LOD) in >16% of the samples and were excluded from further analyses. The majority (*n* = 122; 92.4%) of metabolites were detected in all samples; the 8 remaining metabolites were missing in at most 6.1% of the samples. Our dataset comprised 40 acylcarnitines, 34 lipids (14 lysophospholipids, 10 sphingolipids, and 10 phosphatidylcholines), 24 amino acids, 16 organic acids, 12 biogenic amines, and 4 metabolites classified as ‘other’ (creatine, choline, trimethylamine N-oxide [TMAO], and glucose). Metabolomic data that were used in these analyses can be found in S1 Data.

### Effect of simulated night shift protocol on plasma metabolites

**Fig 1:**
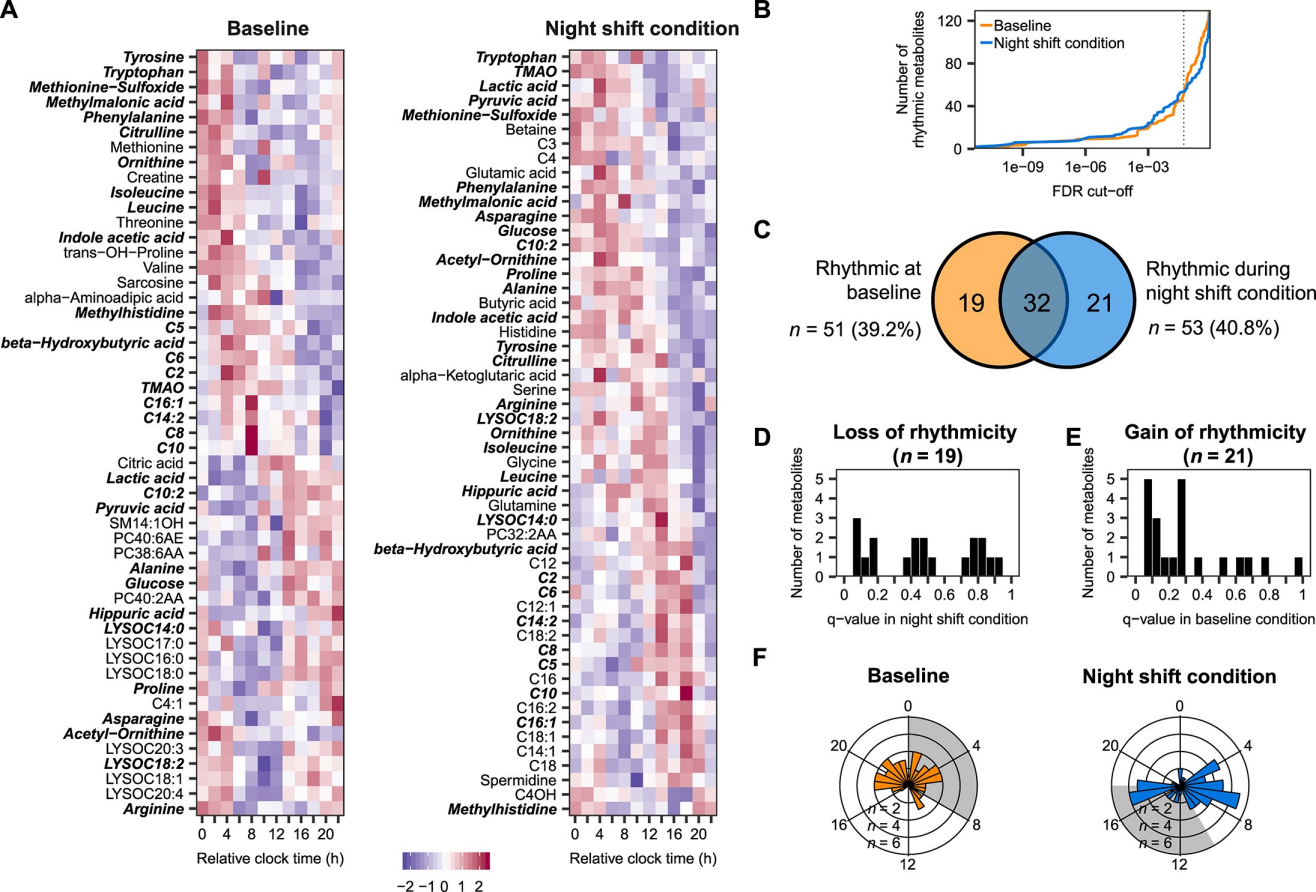
Identification of metabolite rhythms at baseline and during the night shift condition. (A) Heatmap of metabolites identified as rhythmic by mixed-effects cosinor analysis (FDR < 0.05) at baseline (left) and during the night shift condition (right). Metabolites that are rhythmic in both conditions are shown in bold-italic. (B) Number of rhythmic metabolites across the continuum of FDR cutoffs. Dotted vertical line represents an FDR cutoff of 0.05. (C) Venn diagram depicting the number of metabolites identified as significantly rhythmic at baseline, during the night shift condition, and the overlap. (D, E) Histogram displaying the distribution of q-values obtained by mixed-effects cosinor analysis on (D) the 19 metabolites that were rhythmic at baseline but not during the night shift condition and (E) the 21 metabolites that were rhythmic during the night shift condition but not at baseline. (F) Phase distribution of metabolites identified as rhythmic at baseline (left) and during circadian misalignment (right). Shaded areas represent the scheduled sleep periods in the two conditions. Numerical data underlying the results presented in this figure are available in S2 Data. FDR, false discovery rate; TMAO, trimethylamine N-oxide.

Using mixed-effects cosinor analysis on data collected during the baseline and on the night shift condition separately, we identified 51 metabolites (39.2% of the total metabolites on the platform) that showed a significant 24-h rhythm at baseline (false discovery rate [FDR] < 0.05) and 53 metabolites (40.8%) that were rhythmic during the night shift condition (Fig 1A). Across the continuum of FDR cutoffs, the number of rhythmic metabolites was largely similar at baseline and during the night shift condition (Fig 1B), confirming the comparable numbers in the two conditions observed at our selected FDR cutoff. A significant overlap was detected between the metabolites that were identified as significantly rhythmic at baseline and during the night shift schedule (*p* < 0.0001, hypergeometric test), with 32 metabolites identified as rhythmic in both conditions (Fig 1C). The 19 metabolites that were rhythmic at baseline but not during the night shift condition predominantly consisted of lipids (6 lysophospholipids, 3 phosphatidylcholines, and 1 sphingolipid). Class overrepresentation analysis revealed that lysophospholipids were significantly enriched among this list of metabolites that lost their rhythms during the night shift condition, while other classes were not (S1A Table). The 21 metabolites that were rhythmic during the night shift condition but not at baseline were significantly enriched for acylcarnitines (S1B Table). We next explored whether the metabolites that lost or gained rhythmicity during the night shift condition went from robustly rhythmic in one condition to either no discernible rhythm or to a weaker rhythm that did not meet the FDR cutoff by a small margin in the other condition. Interestingly, q-values of metabolites that lost their rhythms were uniformly distributed across the entire range of q-values (Fig 1D). However, the q-value distribution of metabolites that gained rhythmicity during the night shift condition was more skewed towards lower q-values (Fig 1E).This indicates that many of the metabolites that were identified as rhythmic during the night shift condition already showed signs of rhythmicity at baseline, but did not meet our stringent FDR cutoff. We subsequently determined the distribution of phases (i.e., times of highest concentration, as determined by cosinor analysis) of rhythmic metabolites and found that these were nonuniformly distributed throughout the 24-h period both at baseline and during the night shift condition (both *p* < 0.05; Rao spacing test; Fig 1F). At baseline, rhythmic metabolites peaked throughout the 24-h period, except for a 5-h gap 11–16 h after the onset of the habitual sleep period, whereas during the night shift condition, a bimodal phase distribution was observed, with two subsets of metabolites peaking approximately 7 h and 17 h after the onset of the habitual sleep period. Of note, a subset of the rhythmic metabolites identified in our study were also identified as rhythmic by Skene and colleagues [19], with a significant correlation between the phases of metabolites that were rhythmic in the night shift condition of both studies, but not during the control conditions (S2 Fig).

In addition, using linear mixed-effects modelling to assess overall metabolite levels, we identified 27 metabolites that had significantly different levels during the night shift condition compared with baseline: levels were significantly increased for 7 metabolites (4 lysophospholipids, hippuric acid, spermidine, and threonine) and significantly decreased for 20 metabolites (6 acylcarnitines, 4 organic acids, 3 amino acids, 3 biogenic amines, 2 sphingolipids, TMAO, and choline), with the direction of change largely consistent across subjects (S3 Fig). Class overrepresentation analysis revealed significant enrichment of lysophospholipids among the metabolites that had increased levels during the night shift condition (S1C Table). No significantly enriched classes were found among the metabolites with decreased levels during the night shift condition (S1D Table).

### Identification of circadian- and behaviour-influenced metabolites

**Fig 2:**
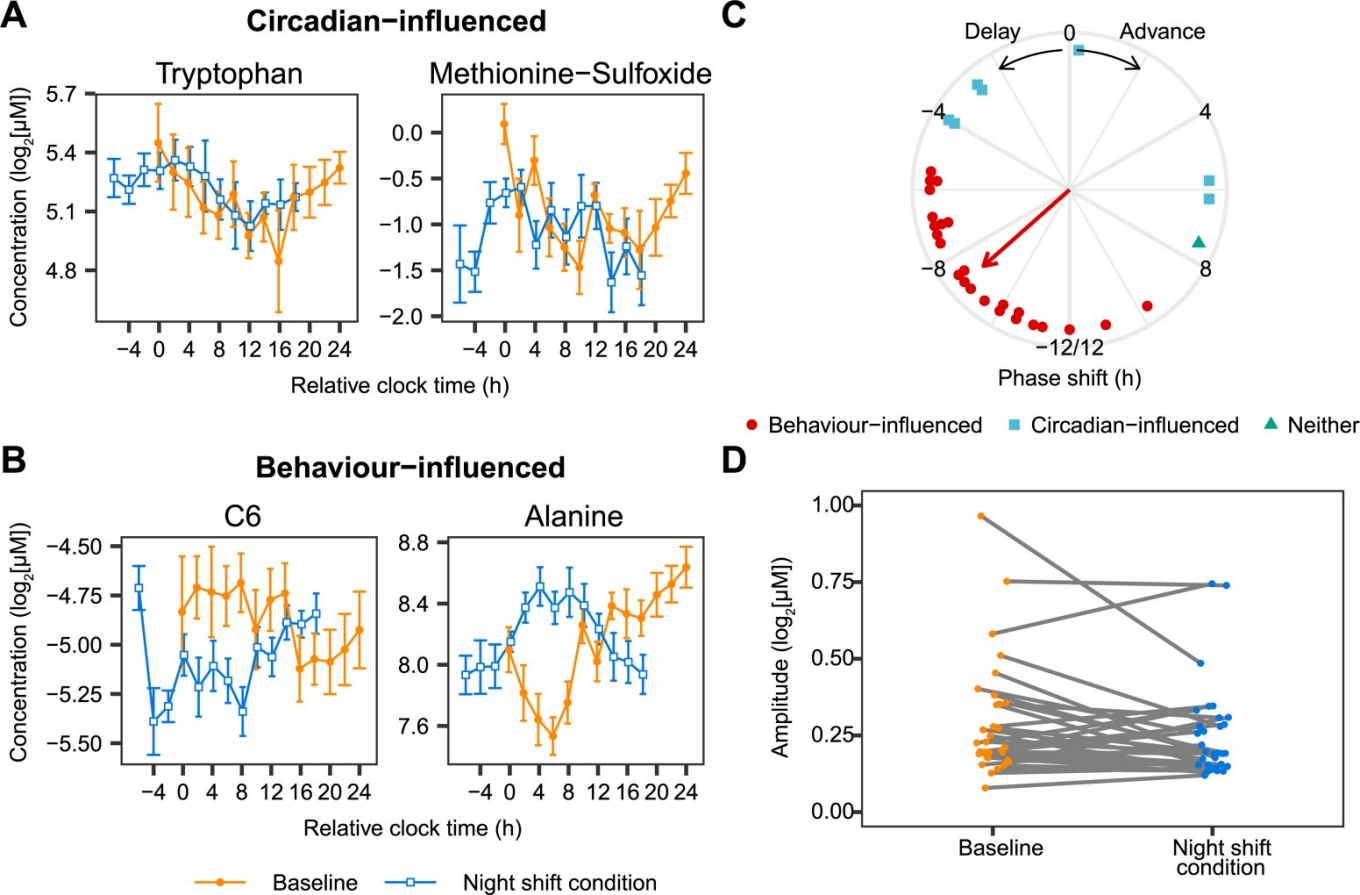
Identification of circadian- and behaviour-influenced metabolites. (A, B) Examples of metabolites that are influenced by the circadian cycle (A: tryptophan and methionine sulfoxide) and that are influenced by the behavioural cycle (B: hexanoylcarnitine [C6] and alanine). Data are presented as mean ± SEM. Relative clock time reflects the time since the start of the individual habitual rest period during baseline. (C) Phase shift during the night shift condition relative to baseline of the 32 metabolites that are significantly rhythmic at both baseline and during the night shift condition. Symbols represent whether the metabolites were classified as circadian-influenced, behaviour-influenced, or neither. The direction of the arrow represents the average phase shift of the behaviour-influenced metabolites. Length of the arrow represents the mean resultant vector length, a measure of the spread of the circular data. No arrow is shown for the circadian-influenced metabolites, because the phase shift of the metabolites was not nonuniformly distributed (*p* = 0.16, Rayleigh test). (D) Amplitude of the 32 metabolites that were identified as rhythmic during baseline and the night shift condition. Numerical data underlying the results presented in this figure are available in S2 Data. C6, hexanoylcarnitine.

Next, we investigated whether the 32 metabolites rhythmic in both conditions were either influenced by the circadian cycle or by behavioural cycles of fasting-feeding and sleeping-waking using a model selection approach (see Materials and methods for details). Twenty-four metabolites were identified as behaviour-influenced, while 7 were identified as circadian-influenced. One metabolite, the lysophospholipid lysoPC(14:0), was classified in neither category. Examples of the circadian- and behaviour-influenced metabolites are shown in Fig 2A and 2B; a full list of the metabolites and their classification can be found in S3 Data. A Rayleigh test showed that the phase shifts of the 24 behaviour-influenced metabolites were not uniformly distributed around the 24-h cycle (*p* < 0.0001) and had an average phase delay of 8.8 ± 0.62 h (circular mean ± SD), consistent with the 10-h delay of the behavioural cycles (Fig 2C). The phase shifts of the circadian-influenced metabolites were not significantly nonuniformly distributed around the 24-h cycle (*p* = 0.16, Rayleigh test). In addition, the amplitude of these metabolites was not affected by the night shift condition (baseline: 0.22 ± 0.18; night shift condition: 0.19 ± 0.14 [median ± IQR]; *p* = 0.172; paired Wilcoxon signed rank test; Fig 2D). Overrepresentation analysis revealed that amino acids were significantly overrepresented among the 24 behaviour-influenced metabolites (S1E Table), while no metabolite classes were enriched among the circadian-influenced metabolites (S1F Table).

### Individual differences in the response to the simulated night shift protocol

**Fig 3:**
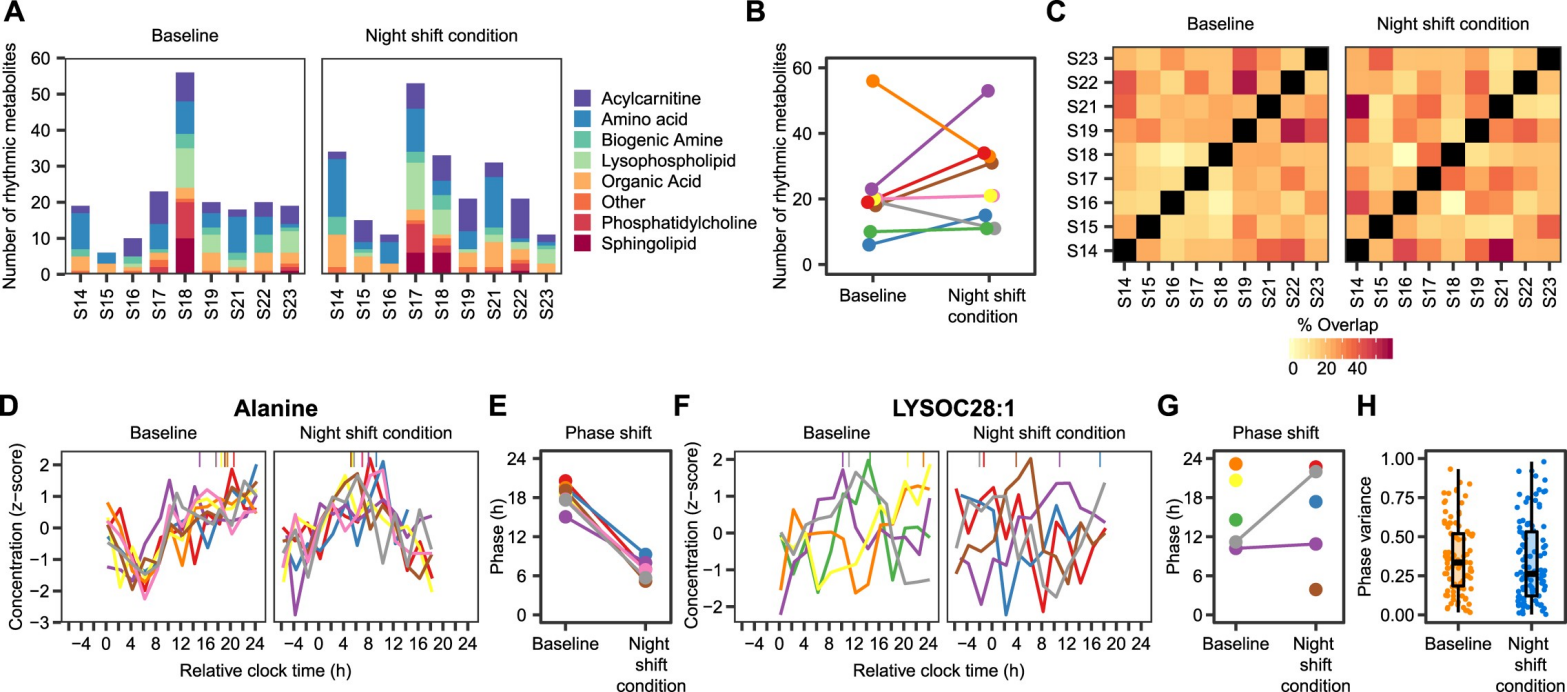
Individual variability in metabolite rhythms. (A) Number of significantly rhythmic metabolites (corrected *p* < 0.05, individual cosinor analysis) per metabolite class and per subject at baseline (left panel) and in the night shift condition (right panel). (B) Change in number of rhythmic metabolites at baseline and during the night shift condition. Different lines and colours represent different subjects. (C) Percentage of rhythmic metabolites that overlap between each pair of subjects. (D, F) Rhythmic alanine (D) and LysoC28:1 (F) profiles per subject at baseline and the night shift condition. Symbols on top of each panel indicate the phase of the rhythm. Different colours represent different subjects. Only rhythmic profiles (uncorrected *p* < 0.05) are shown. (E, G) Initial and final phases of alanine (E) and LysoC28:1 (G) profiles per subject. Colours, representing different subjects, matched with the colours in B, D, and F. (H) Circular variance of individual phase estimates per metabolite at baseline and during the night shift condition. Circular variance ranges from 0 to 1, with lower values indicating tighter clustering of the phase estimates around the mean and higher values indicating a higher degree of dispersion around the 24-h cycle. Only metabolites that were rhythmic (uncorrected *p*-value <0.05) in at least 3 subjects were taken into account. Numerical data underlying the results presented in this figure are available in S2 Data. LysoC, lysophospholipid.

To characterise interindividual differences in 24-h rhythms in the metabolome, cosinor analysis was performed on individual metabolite profiles to assess 24-h rhythmicity on an individual level. The number of significantly rhythmic metabolites (corrected *p*-value <0.05) per subject at baseline or during the night shift condition ranged from 6 to 56 and represented all classes of metabolites on the platform (Fig 3A). The large degree of interindividual variability in the number of rhythmic metabolites was preserved across different *p*-value cutoffs (S4 Fig). However, no effect of simulated night shift protocol on the number of rhythmic metabolites was found (Fig 3B). The median number of rhythmic metabolites was 19 (range: 6–56) at baseline and 21 (range: 11–53) during the night shift condition (*p* = 0.235, paired Wilcoxon signed rank test). Likewise, the overlap of rhythmic metabolites between subjects was not affected by the night shift protocol (Fig 3C), with a median overlap of 21% (range: 3.0%–55%) at baseline and 22% (range: 0%–59%) during the night shift schedule (*p* = 0.614, paired Wilcoxon signed rank test).

Inspection of the individual metabolite profiles revealed extensive interindividual variability in the phases of rhythmic metabolites. For example, rhythmic alanine profiles (uncorrected *p*-value <0.05) were highly synchronous at both baseline and during the night shift condition (both *p* < 0.001, Rayleigh test; Fig 3D), with the peak occurring during the wake period at baseline and during the night shift condition (Fig 3E). In contrast, the phases of rhythmic lysoPC(28:1) profiles (Fig 3F) were dispersed throughout the 24-h period during both baseline and the night shift condition (both *p* > 0.69, Rayleigh test; Fig 3G). In general, the circular variance of the individual phase estimates per metabolite revealed a high degree of heterogeneity across subjects, depending on the metabolite, but did not differ between baseline and the night shift condition (*p* = 0.2579, Wilcoxon signed rank test; Fig 3H).

**Fig 4:**
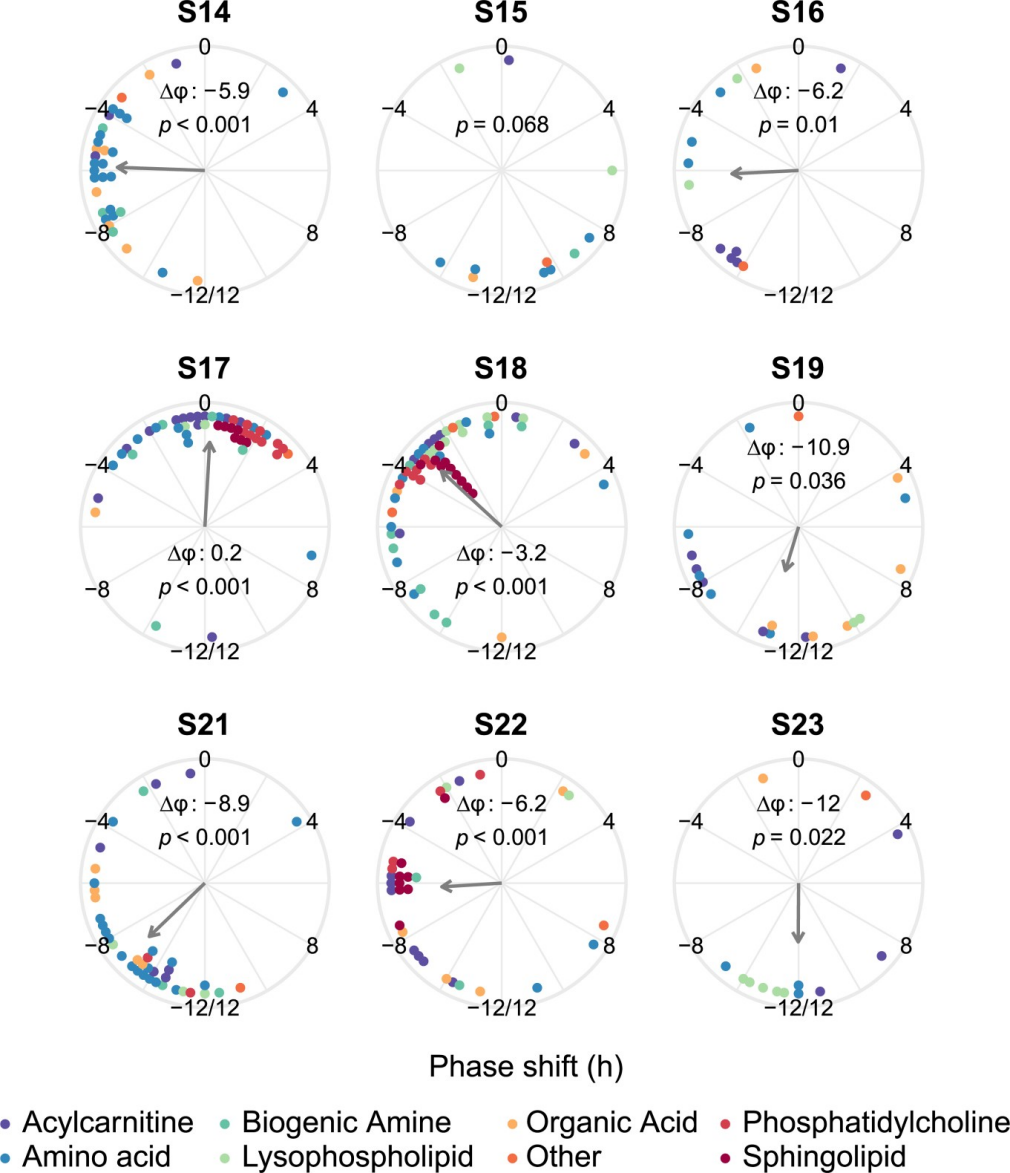
Phase shifts of rhythmic metabolites per subject. Panels show the data for each of the 9 subjects. Dots represent the phase shifts of metabolites that are rhythmic (uncorrected *p* < 0.05) both at baseline and during the night shift schedule in that subject. By convention, a negative phase shift denotes a phase delay; a positive phase shift denotes a phase advance. The *p*-value for the significance of the mean direction of the phase shift (Rayleigh test) is shown in each panel, as well as the average phase shift (Δφ) if the Rayleigh test *p*-value is <0.05. Arrow length represents the resultant vector length, an indication of the strength of the mean direction. The colour of the dots represents the metabolite class, as indicated in the legend. Numerical data underlying the results presented in this figure are available in S2 Data.

We next computed the phase shifts of metabolites that were rhythmic (*p* < 0.05) at baseline and during the night shift condition within each subject. In all but one subject the phase shifts of the metabolites were nonuniformly distributed, with a significant mean direction (Fig 4). The average phase shifts ranged from a 0.2-h advance in one subject to a 12.0-h delay in another subject (Fig 4). The mean direction of the average phase shifts (a delay of 6.8 h) was not significant (*p* = 0.0508, Rayleigh test). It should be noted that the *p*-value was close to 0.05, indicating that more research is required to determine whether the average phase shifts are uniformly distributed around the 24-h cycle or, alternatively, point in a specific direction. Altogether, these results suggest that the phases of rhythmic metabolites shift in a specific direction within most subjects, but that a large degree of differences exist regarding the direction of this phase shift across subjects. The individual estimates of phases and phase shifts were significantly correlated with the group-level phases and phase shifts of rhythmic metabolites (all *p* < 0.001, circular version of the Pearson product-moment correlation; S5 Fig). This indicates that the group-level analysis still reflected the average phase-shifting behaviour of metabolites in individual subjects, despite the large degree of heterogeneity among subjects.

## Discussion

A detailed characterisation of the metabolic processes that are impacted by night shifts and the concomitant displacement of sleep and food intake is required to better understand the increased risk of adverse metabolic effects observed in shift workers. Here, we used a targeted metabolomics approach to determine the impact of a simulated night shift protocol on 24-h rhythms in circulating metabolite levels. Overall, our results demonstrated that 75% of rhythmic metabolite profiles are primarily influenced by behavioural cycles, such as fasting-feeding and sleeping-waking cycles, rather than by the endogenous circadian clock. Hence, on the group level, the majority of rhythmic metabolites had phase shifted in response to the night shift condition, leading to a state of misalignment relative to the endogenous circadian system. Our within-subject study design allowed us to also analyse the interindividual variability in the effect of the simulated night shift protocol, revealing a high degree of heterogeneity across subjects, but a stable pattern within subjects regarding the phase shift of rhythmic metabolites. Altogether, our study provides novel insight into the effect of circadian misalignment, as occurs during night shift work, on the temporal coordination of the human plasma metabolome and sheds light on the individual differences in this response.

Using a model selection approach to statistically assess which metabolites are driven by behavioural versus circadian cycles, we found that a large majority (24 out of 32; 75%) of metabolites that are rhythmic in both experimental conditions are influenced by the shifted behavioural cycles rather than by the endogenous circadian clock, showing an average phase delay of 8.8 h. Overrepresentation analysis revealed that amino acids were significantly enriched among these 24 metabolites. It has been reported previously that metabolites involved in amino acid–related processes exhibit a circadian rhythm in mice and humans independent of sleep-wake and fasting-feeding cycles [20]. However, our findings suggest that the shift of the behavioural cycles drives the rhythms in the levels of amino acids during circadian misalignment. In light of our previous findings that the phase of central circadian clock markers, peripheral clock gene expression, and transcriptome-wide gene expression rhythms do not shift in response to the night shift condition [21,22], the extensive regulation of metabolite rhythms by behavioural cycles suggests that the phase angle between different physiological rhythms is disturbed. This potentially induces a state of desynchrony between the temporal coordination of the plasma metabolome and transcriptionally driven circadian clocks. However, it should be noted that the levels of metabolites in blood are influenced by metabolic processes across different tissues [23], while gene expression levels were measured locally in peripheral blood mononuclear cells, which precludes a direct evaluation of the relationships between metabolites and transcriptional rhythms in blood.

The large proportion of metabolites that are driven by behavioural cycles is consistent with studies in mice showing that imposed feeding rhythms drive daily rhythms in peripheral metabolism subjected to time-restricted feeding [24,25]. In addition, our results corroborate prior observations by Skene and colleagues (2018) that the phases of 95% of the human blood metabolome shift significantly during a night shift condition compared with a day shift condition [19]. Metabolites that were identified as rhythmic in both our study and that of Skene and colleagues (2018) included mainly amino acids and lysophospholipids in the control conditions and amino acids and some acylcarnitines in the night shift condition. In addition, we found a significant enrichment of lysophospholipids among the metabolites that lost their rhythm during the night shift condition, which is consistent with the results presented by Skene and colleagues (2018), who report that 7 out of 19 metabolites that are only rhythmic during the day shift condition are lysophospholipids. To further compare the two studies, we compared the phases of the commonly rhythmic metabolites, although it should be noted that Skene and colleagues (2018) use actual clock times, whereas we report time relative to the subjects’ individual habitual bedtimes in order to account for interindividual differences in sleep timing. We found a significant correlation between the phases of rhythmic metabolites for the night shift condition, but not for the control condition. The lack of correlation between the phases of metabolites during the control conditions may be due to the experimental designs, which differed mainly in terms of the control condition that was used: we used a within-subject design in which participants were studied at baseline and on the fourth day of the simulated night shift protocol, allowing us to address interindividual variability in the effect of circadian misalignment on the metabolome, whereas Skene and colleagues (2018) used a between-subjects design in which separate groups of participants were studied after three days on a day shift schedule or a night shift schedule. Altogether, our findings and those presented by Skene and colleagues (2018) indicate that metabolite rhythms are predominantly influenced by behavioural cycles.

Seven metabolites were classified as circadian-influenced. Instead of shifting along with the behavioural cycles like the majority of metabolites, the rhythms in the levels of these metabolites remained aligned with the non-shifted melatonin phase [26], which is typically used as a marker of the phase of the central circadian clock [27]. Therefore, the levels of these metabolites do not seem to be driven by the imposed sleep-wake and fasting-feeding cycles, but may rather be regulated by the non-shifting central or peripheral clocks. This creates a state of misalignment between these 7 metabolite rhythms and the shifted sleep-wake and fasting-feeding rhythms. Interestingly, among these metabolites, 2 are related to the pathway involved in melatonin synthesis: tryptophan and indole-acetic acid. However, it should be noted that the rate-limiting enzyme in melatonin biosynthesis is downstream of these 2 metabolites [28], so it is unlikely that the observed metabolite rhythms drive the melatonin rhythm.

In field studies involving shift workers, it is well known that there are considerable individual differences regarding the extent of adaptation of the central and peripheral circadian clocks to night shift work [5,29–31], but individual differences in adaptation of 24-h rhythms in other physiological processes are largely unexplored. Therefore, we sought to probe the extent of interindividual variability in the response of the metabolome to the simulated night shift protocol, which is made possible by the within-subject design of our study. Firstly, we found that there is a large degree of interindividual variability regarding the number of rhythmic metabolites, which ranged from 4.6% to 43%. The set of metabolites that were rhythmic across subjects also differed considerably: the median overlap between pairs of subjects was 21% during baseline and 22% during the night shift condition. These numbers are similar to what was previously reported in a laboratory study investigating the individual differences in rhythmic lipid species during a constant routine protocol in healthy subjects, in which a median agreement of 20% of all pairwise comparisons between subjects was found [32]. Previous studies also reported extensive diversity in the timing of metabolite rhythms between subjects [32,33]. Our study extends these findings by revealing substantial differences between subjects regarding the phase-shifting response of metabolite rhythms to the simulated night shift protocol: while a significant concentration of rhythmic metabolite phase shifts was found in 8 out of 9 subjects, as opposed to a uniform distribution, the direction of these phase shifts was highly heterogeneous, ranging from a 0.2-h advance to a 12.0-h delay, suggesting the existence of an individual metabolomic signature of circadian misalignment. In general, the large extent of interindividual differences in the 24-h rhythms in the metabolome presented here and in previous studies [32,33], as well as the individual response to circadian misalignment as shown in this study, warrants more attention in future studies.

Our results also indicated that the overall levels of 27 metabolites (21% of the measured metabolites) significantly changed in response to a night shift schedule. We found a significant overrepresentation of lysophospholipids among the metabolites with increased levels, suggesting altered lipid metabolism in response to the night shift condition. Furthermore, it is interesting to note that this included 4 metabolites involved in methionine metabolism: betaine, choline, and sarcosine levels were significantly decreased, while spermidine levels were significantly increased. Methionine metabolism plays a crucial role in epigenetics by generating S-adenosylmethionine, which provides the methyl group used for histone and DNA methylation [34]. In general, fluctuations in the levels of metabolites involved in this process within the physiological range can alter histone methylation and, as a consequence, gene transcription [35]. Prior research has shown that interventions that affect the circadian system, such as altered light-dark cycles or sleep restriction, can influence epigenetic regulation [36–38]. Therefore, further research is required to investigate the potential effects of altered metabolite levels during circadian misalignment on epigenetic modifications.

An asset of our study is the use of targeted metabolomic profiling, which allowed us to acquire quantitative data of a predetermined set of metabolites [39]. However, it should be noted that the use of an untargeted approach would have allowed us to identify additional metabolites and pathways that are altered as a result of the night shift condition. An aspect of this study that could be considered a limitation is the measurement of metabolite concentrations in plasma, rather than in specific tissues. Although the measurement of metabolomic profiles in blood provides an accessible window into the biochemical changes that occur in the entire body during different nutritional, pathophysiological, or environmental conditions [40], daily variations in metabolite levels have been shown to be highly tissue specific [41,42]. On the other hand, similar metabolomic changes were found in serum and liver of mice exposed to chronic jet lag [43]. Therefore, future studies should address the tissue-specific response to circadian misalignment, although this will be challenging in humans due to the invasive nature of such experiments. Another limitation of this study is that the food intake was not optimally controlled: during the 16-h waking portion of the measurement periods, subjects consumed hourly isocaloric snacks, while they fasted during the 8-h sleep period. This is a slightly different approach than that used in a prior study into the effect of the circadian and behavioural cycles on the metabolome, in which samples were collected from subjects consuming isocaloric snacks during a 24-h constant routine procedure [19]. However, the similarities between our findings and that study, as discussed above, reinforce the reliability of our findings. Furthermore, our sample size was relatively small and consisted of healthy subjects with limited demographic diversity. Sex differences in the effect of circadian misalignment on the metabolome also remain to be addressed. Future studies should be conducted in subjects with a wider age range and in different populations, including actual night shift workers. This will help to determine the factors that contribute to the interindividual variation in the 24-h rhythms in the metabolome and that could potentially be used to predict an individual’s metabolic response to circadian misalignment.

In conclusion, our results provide insight into the effects of a simulated night shift protocol on the 24-h variation of metabolic processes in humans. We found that, on the group level, 24-h rhythms in metabolite levels are primarily driven by the fasting-feeding and sleep-wake cycles. However, taking advantage of our within-subject design, we were able to examine the individual responses to the simulated night shift protocol. Importantly, we found extensive interindividual variability in the response to such a protocol, with metabolite rhythms largely adapting to the shifted behavioural cycles in some subjects, but no evidence of adaptation in other subjects. This suggests that circadian misalignment has an individual metabolomic signature. As such, these findings highlight the importance of taking into account individual responses to circadian misalignment, which is highly relevant given the variability in adaptation to shifted schedules among shift workers [5,29]. Altogether, our study contributes to the understanding of the adverse metabolic effects of circadian misalignment, as occurs during night shift work. Further studies are required to examine these effects in actual night shift workers and in other populations at risk of experiencing circadian misalignment.

## Materials and methods

### Ethics statement

All subjects provided written informed consent prior to the study. The study was approved by the Douglas Institute Ethics Board (protocol number: IUSMD-03-32) and was conducted according to the Declaration of Helsinki.

### Study design

Ten healthy young adults (9 men, 1 woman) were enrolled in a simulated night shift work protocol. Details on the recruitment, screening, and experimental protocol were previously published [22,26]. One male subject only completed the baseline session, and his results were excluded from all analyses.

For at least 1 wk prior to admission to the laboratory, subjects maintained a stable, self-selected sleep-wake schedule with a sleep period of 8 h. Sleep diaries and actigraphy were used to assess compliance. The laboratory protocol involved a 24-h baseline measurement period, during which subjects slept according to their habitual sleep-wake schedule, remained in a constant posture to keep activity levels to a minimum, consumed hourly isocaloric snacks, and were exposed to dim light (<10 lux) (S1 Fig). The caloric requirement was calculated per subject based on the Harris and Benedict formula using an activity factor of 1.3 [44]. On the following days, the sleep period was delayed by 10 h relative to the habitual sleep period. Subjects were exposed to dim light levels throughout all wake periods during the entire study. On the fourth day of this night shift schedule, subjects underwent a second 24-h measurement period under similar conditions as the baseline measurement period (minimal activity levels, constant posture, hourly isocaloric snacks, exposure to dim light). Blood samples (10 mL) for metabolomic analysis were collected in heparin-coated tubes every 2 h during both measurement periods via an indwelling catheter. Plasma was separated through centrifugation for 30 min at 370*g* on a density gradient (Histopaque-1077, Sigma Aldrich, Oakville, Canada) and stored at −80°C until further processing.

### LC-MS/MS analysis method

Targeted quantitative metabolomics was used to analyse the plasma samples using a combination of direct injection mass spectrometry (DI-MS) with a reverse-phase LC-MS/MS assay. The method combines the derivatisation and extraction of analytes, and selective mass-spectrometric detection using multiple reaction monitoring (MRM) pairs. Isotope-labeled internal standards were used for metabolite quantification. All plasma samples were thawed on ice, vortexed, and centrifuged at 13,000*g*. Ten microliters of each plasma sample were loaded and dried in a stream of nitrogen. Then, 20 μL of a 5% solution of phenyl-isothiocyanate was added for derivatisation. After incubation, the samples were dried again using an evaporator. Extraction of the metabolites was then achieved by adding 300 μL methanol containing 5 mM ammonium acetate. For organic acid analysis, internal standards and ice-cold methanol were added to the plasma samples. Next, the samples were vortexed and incubated at 4°C. Samples were stored at −20°C for protein precipitation. After protein precipitation, serum samples were centrifuged. EDC, 3-NPH, and 7.5% pyridine in methanol were added to the supernatants. The samples were incubated at room temperature, followed by addition of HPLC water and BHT in methanol. Mass spectrometric analysis was performed on an API4000 Qtrap tandem mass spectrometry instrument (Applied Biosystems/MDS Analytical Technologies, Foster City, CA) equipped with a solvent delivery system. The samples were delivered to the mass spectrometer by an LC method followed by a direct injection method. Concentrations were calculated using Analyst software (AB Sciex, Concord, Canada). Detected metabolites were included for further analysis if they were above the LOD in at least 90% of the samples. Values below the LOD were set to 50% of the lowest detected value. All downstream analysis was performed on log_2_-transformed values in R v3.5.1. [45] (S1 Data).

### Group-level rhythmicity analysis

To assess rhythmicity of metabolite levels on the group level at baseline and during the night shift condition separately, mixed-effect linearised cosinor analysis [46] was performed using the R package lme4 [47] as described previously [22]. The subject was included as a random effect on the mesor. For each metabolite, the following statistical model was fit to all data available from the baseline condition and then separately on all data available from the night shift condition:

$$y_{ijk}=a_{k}+b_{k}*\cos\left(\frac{2\pi *t_{ij}}{24}\right)+c_{k}*\sin(\frac{2\pi *t_{ij}}{24})+\eta_{ik}+\varepsilon_{ijk}$$ Model 1

In this model, *y*_*ijk*_ is the log_2_-transformed concentration of metabolite *k* in individual *i* at time point *j*, *a*_*k*_ is the intercept (mesor), *b*_*k*_ and *c*_*k*_ are cosinor coefficients, *t*_*ij*_ is the sampling time in hours after the onset of the habitual sleep period (i.e., relative clock time), *η*_*ik*_ is the interindividual variability, and *ε*_*ijk*_ is the residual variability. The fit of the model was compared with the null model, in which *b*_*k*_ = *c*_*k*_ = 0, using the likelihood ratio test. The *p*-values were adjusted for multiple testing using the Benjamini-Hochberg method [48]. The phase (i.e., time of peak) of the metabolite rhythms were calculated from the cosinor coefficients [46]. Circular statistics were performed using the R package ‘circular’ v0.4–93 [49].

### Change in overall levels

To assess changes in the overall levels of the metabolites during the night shift condition compared with baseline, linear mixed-effects modelling was performed on each metabolite, with the log_2_-transformed concentrations as the dependent variable, the condition (baseline versus night shift condition) as fixed effect, and the subject as random effect. The *p*-values were adjusted for multiple testing using the Benjamini-Hochberg method [48].

### Identification of circadian- and behaviour-influenced metabolites

To determine whether the metabolites that were rhythmic at baseline and during the night shift condition were influenced by circadian cycles or by behavioural cycles, a model selection approach was used. To this end, two linear mixed-effects models were fit to the baseline and night shift condition data simultaneously: (1) a circadian model and (2) a behavioural model. For the circadian model, Model 1 was fit to all data simultaneously, with *t*_*ij*_ representing the sampling time in hours after the melatonin phase (as reported previously [50]) as a measure of circadian time. The significance of this model was determined by comparing the fit to a null model with *b*_*k*_ = *c*_*k*_ = 0 using the likelihood ratio test (i.e., no rhythmicity). Furthermore, the Bayesian Information Criterion (BIC) was computed. For the behavioural model, Model 1 was fit again to all data simultaneously, now using *t*_*ij*_ as the sampling time in hours after lights off during the protocol as a measure of behavioural time. Again, significance was assessed using the likelihood ratio test, and the BIC was computed. The *p*-values were corrected for multiple testing using the Benjamini-Hochberg method. A metabolite was classified as ‘circadian-influenced’ (1) if the corrected *p*-value of the circadian model was below α = 0.05 and (2) if the BIC of the circadian model was lower than the BIC of the behavioural model (indicating an improved goodness of fit). A metabolite was classified as ‘behaviour-influenced’ (1) if the corrected *p*-value of the behavioural model was below α = 0.05 and (2) if the BIC of the behavioural model was lower than the BIC of the circadian model.

### Individual times series analysis

Cosinor analysis on individual metabolite profiles was performed as previously described [22]. Briefly, *p*-values were derived from a zero-amplitude test [46]. To obtain the number of rhythmic metabolites and the overlap among subjects, *p*-values were corrected for multiple testing using permutations of 1,000 randomly shuffled time series. The phase (and phase shifts) of metabolite profiles were calculated from the cosinor coefficients for all metabolites with uncorrected *p* < 0.05.

### Class overrepresentation analysis

Fisher exact tests were performed to find metabolite classes (i.e., acylcarnitines, amino acids, biogenic amines, lysophospholipids, organic acids, phosphatidylcholines, or sphingolipids) that were overrepresented among metabolites that lost or gained rhythmicity during the night shift condition, metabolites with significantly increased or decreased levels during the night shift protocol, and metabolites that were identified as behaviour influenced. A metabolite class was considered significantly overrepresented if *p* < 0.05.

## Supporting information

S1 Fig Overview of the simulated night shift protocol. After a 24-h blood sampling during a constant posture procedure, the habitual sleep period of the subjects was delayed by 10 h. On the fourth day on this sleep/wake schedule, subjects underwent a second 24-h blood sampling procedure. During the wake episodes of both sampling periods, subjects received hourly isocaloric snacks.
(TIF)

S2 Fig Comparison of rhythmic metabolites identified in our study and in that of Skene and colleagues (2018) [19]. A total of 67 metabolites were shared between the metabolomics platform used in our study and the one used by Skene and colleagues. (A, B) Venn diagrams representing the number of metabolites that were shared across the two platforms and that were identified as rhythmic in our study and by Skene and colleagues, and their overlap during (A) the control condition (baseline in our study and day shift schedule in Skene and colleagues) and (B) the night shift condition. No significant overlap was found between the rhythmic metabolites observed in Skene and colleagues and our study in the control condition (*p* = 0.199, Fisher exact test) and in the night shift condition (*p* = 0.061, Fisher exact test). (C, D) Correlation between the phases of metabolites identified as rhythmic in both our study and that of Skene and colleagues in (C) the control condition and (D) the night shift condition. The phases were not significantly correlated during the control conditions (*p* = 0.884; r = −0.042; circular version of the Pearson product-moment correlation) but were during the night shift conditions (*p* = 0.026; r = 0.646; circular version of the Pearson product-moment correlation). It should also be noted that we used clock time relative to the habitual sleep period of the subjects, whereas Skene and colleagues used actual clock time. Numerical data underlying the results presented in this figure are available in S2 Data.
(TIF)

S3 Fig Significantly increased and decreased metabolite levels following simulated night shifts. Heatmap showing metabolites with significantly altered concentrations during the simulated night shift condition compared with baseline per subject. Data are displayed as z-scored average normalised concentrations per subject per condition. Metabolites are ordered by the magnitude of change (most increased levels during the night shift condition on top to most decreased levels at the bottom). Numerical data underlying the results presented in this figure are available in S2 Data.
(TIF)

S4 Fig Number of rhythmic metabolites per subject across the continuum of q-value cutoffs. The number of rhythmic metabolites per subject is shown at baseline (left) and during the night shift condition (right). Dotted vertical line represents a corrected *p*-value cutoff of 0.05. Different colours represent different subjects (colours match with those in Fig 3). Numerical data underlying the results presented in this figure are available in S2 Data.
(TIF)

S5 Fig Comparison of phase estimates obtained by group-level and individual-level analysis. Coherence of phase estimates (A) and phase shifts (B) derived from the group and individual analyses. (A) Correlation between phase estimates derived from the group cosinor analysis and individual cosinor analysis of the metabolites identified as rhythmic at baseline (*n* = 51) and during the night shift condition (*n* = 53). The average phases of the individual analysis were obtained by computing the circular mean of the phase estimates of all individually rhythmic time series (uncorrected *p*-value <0.05) per metabolite per condition. (B) Correlation between the phase shifts derived from the group cosinor analysis and individual cosinor analysis of the 32 commonly rhythmic metabolites. Phase shifts on the group level were calculated by subtracting the phase during the night shift condition from the phase at baseline for each metabolite. Phase shifts on the individual level were obtained by computing the phase shift per subject per metabolite, after which the average phase shift (circular mean) across subjects was calculated. Only individually rhythmic time series (uncorrected *p*-value <0.05) were used to compute phase shifts per subject. Numerical data underlying the results presented in this figure are available in S2 Data. *p*, significance of the correlation coefficient; R_circ_, circular version of the Pearson product-moment correlation.
(TIF)

S1 Table Results from class overrepresentation analysis. (DOCX)

S1 Data Raw metabolite data, sample information, and metabolite identifiers. Metabolite levels (μM) are log_2_ transformed.
(XLSX)

S2 Data Numerical data behind figures. (XLSX)

S3 Data Categorisation of metabolites as behaviour- or circadian-influenced. (CSV)

## Abbreviations

BIC: Bayesian Information Criterion
DI-MS: direct injection mass spectrometry
FDR: false discovery rate
HPHPA: (3-hydroxyphenyl)-3-hydroxypropionic acid
LOD: limit of detection
lysoC: lysophospholipid
MRM: multiple reaction monitoring
SCN: suprachiasmatic nuclei
TMAO: trimethylamine N-oxide
