# Supplementary figures and images for: Individual metabolomic signatures of circadian misalignment during simulated night shifts in humans
Source: PLoS Biol. 2019 Jun 18;17(6):e3000303. doi: 10.1371/journal.pbio.3000303 (PMC6581237; doi:10.1371/journal.pbio.3000303)

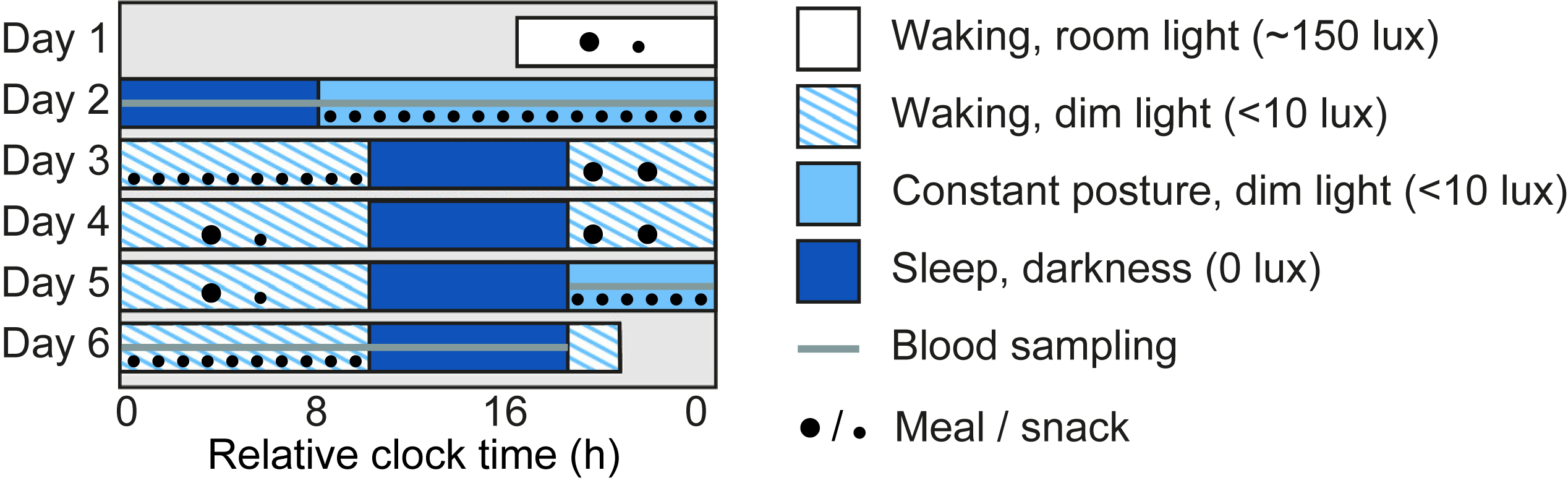

Supplement: S1 Fig — After a 24-h blood sampling during a constant posture procedure, the habitual sleep period of the subjects was delayed by 10 h. On the fourth day on this sleep/wake schedule, subjects underwent a second 24-h blood sampling procedure. During the wake episodes of both sampling periods, subjects received hourly isocaloric snacks. (TIF) [file pbio.3000303.s001.tif]

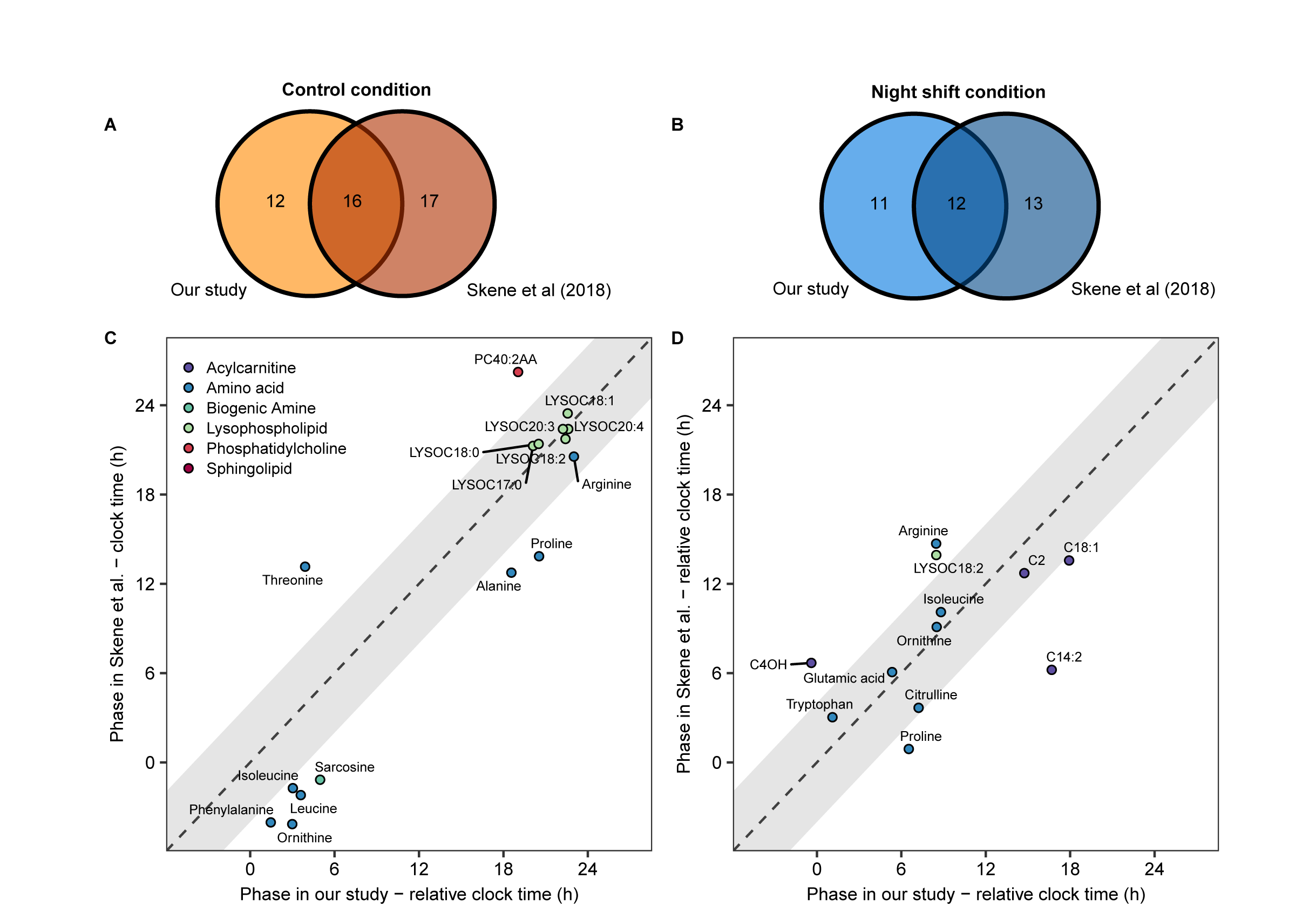

Supplement: S2 Fig — A total of 67 metabolites were shared between the metabolomics platform used in our study and the one used by Skene and colleagues. (A, B) Venn diagrams representing the number of metabolites that were shared across the two platforms and that were identified as rhythmic in our study and by Skene and colleagues, and their overlap during (A) the control condition (baseline in our study and day shift schedule in Skene and colleagues) and (B) the night shift condition. No significant overlap was found between the rhythmic metabolites observed in Skene and colleagues and our study in the control condition (p = 0.199, Fisher exact test) and in the night shift condition (p = 0.061, Fisher exact test). (C, D) Correlation between the phases of metabolites identified as rhythmic in both our study and that of Skene and colleagues in (C) the control condition and (D) the night shift condition. The phases were not significantly correlated during the control conditions (p = 0.884; r = −0.042; circular version of the Pearson product-moment correlation) but were during the night shift conditions (p = 0.026; r = 0.646; circular version of the Pearson product-moment correlation). It should also be noted that we used clock time relative to the habitual sleep period of the subjects, whereas Skene and colleagues used actual clock time. Numerical data underlying the results presented in this figure are available in S2 Data. (TIF) [file pbio.3000303.s002.tif]

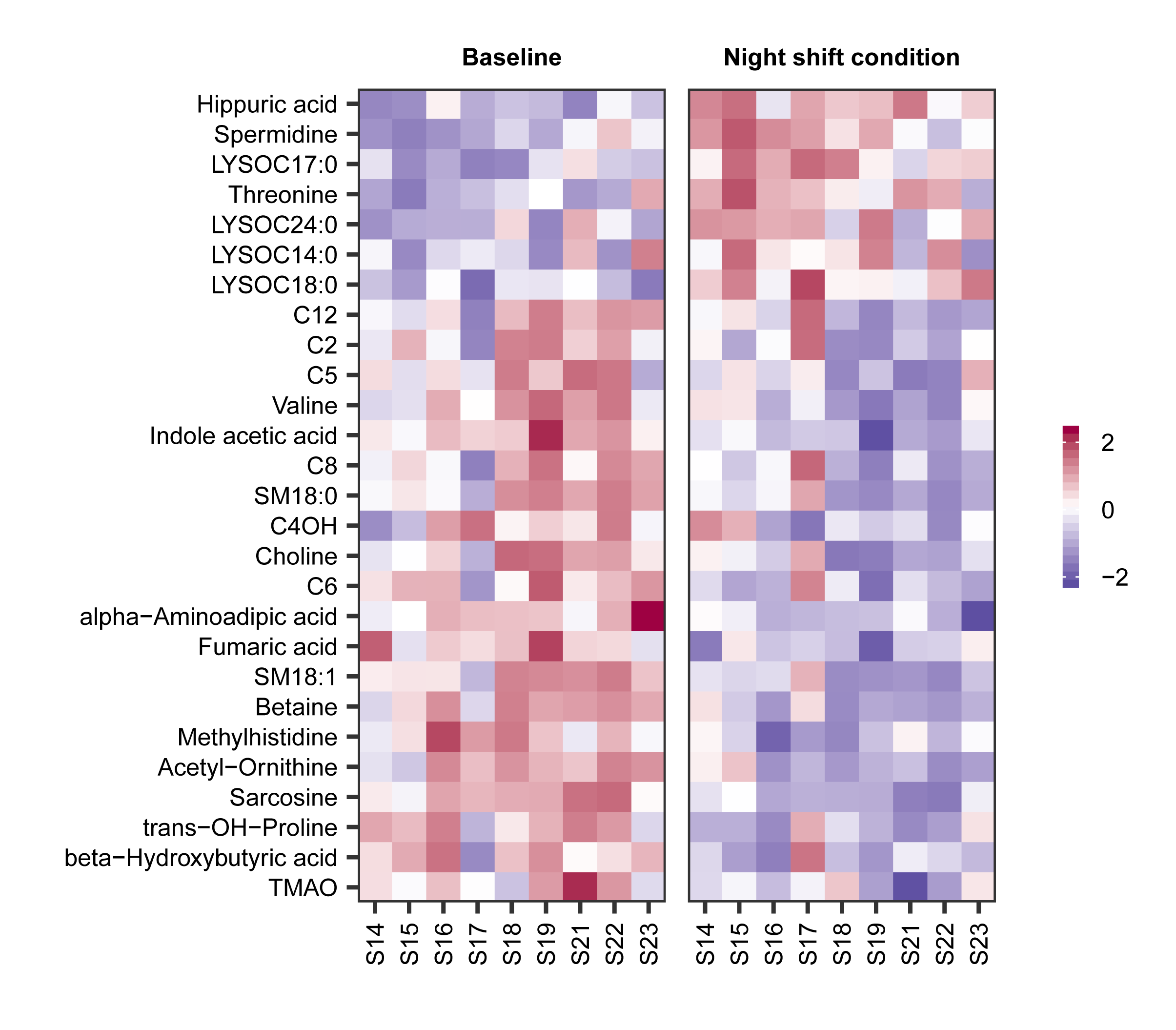

Supplement: S3 Fig — Heatmap showing metabolites with significantly altered concentrations during the simulated night shift condition compared with baseline per subject. Data are displayed as z-scored average normalised concentrations per subject per condition. Metabolites are ordered by the magnitude of change (most increased levels during the night shift condition on top to most decreased levels at the bottom). Numerical data underlying the results presented in this figure are available in S2 Data. (TIF) [file pbio.3000303.s003.tif]

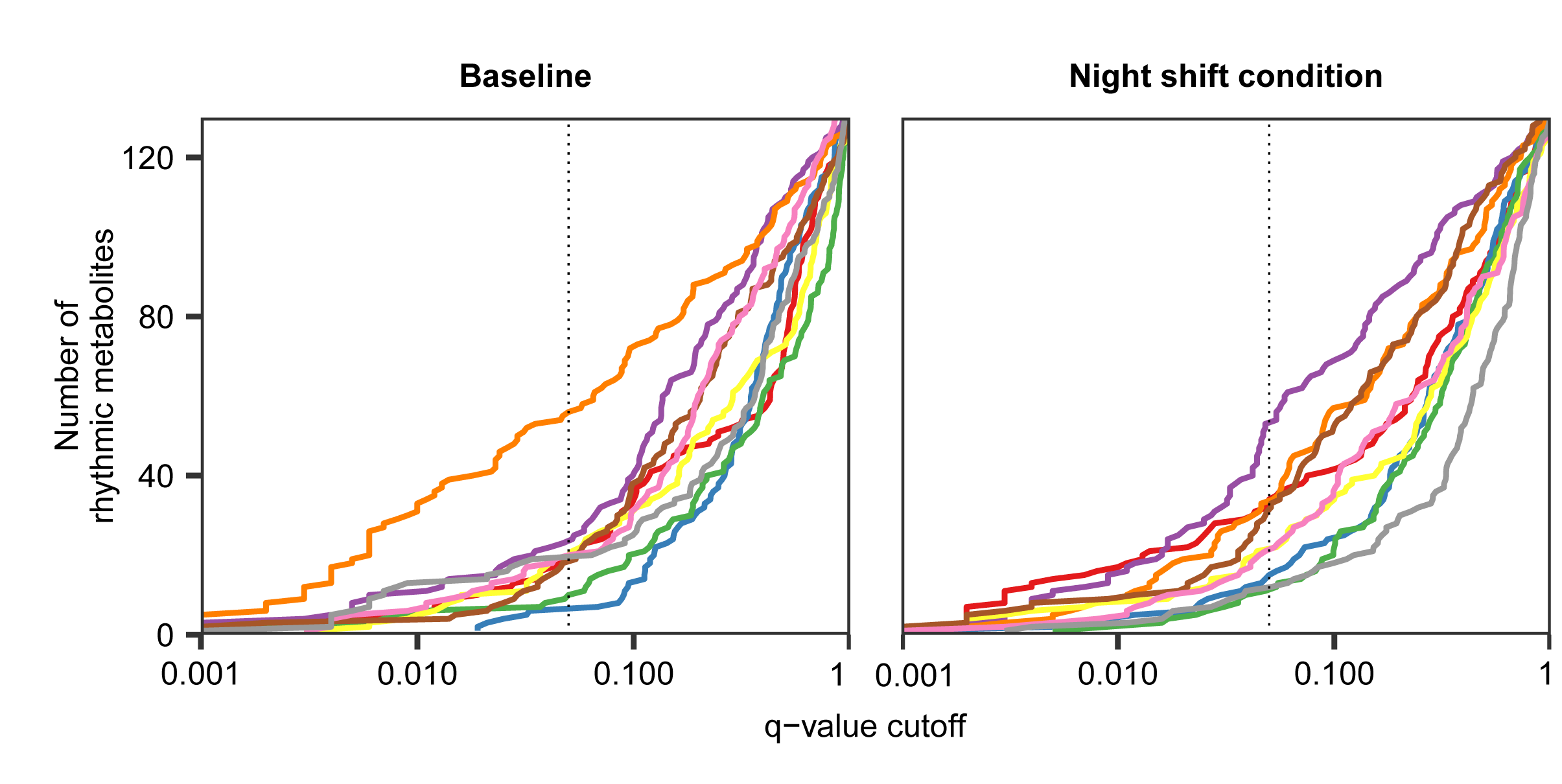

Supplement: S4 Fig — The number of rhythmic metabolites per subject is shown at baseline (left) and during the night shift condition (right). Dotted vertical line represents a corrected p-value cutoff of 0.05. Different colours represent different subjects (colours match with those in Fig 3). Numerical data underlying the results presented in this figure are available in S2 Data. (TIF) [file pbio.3000303.s004.tif]

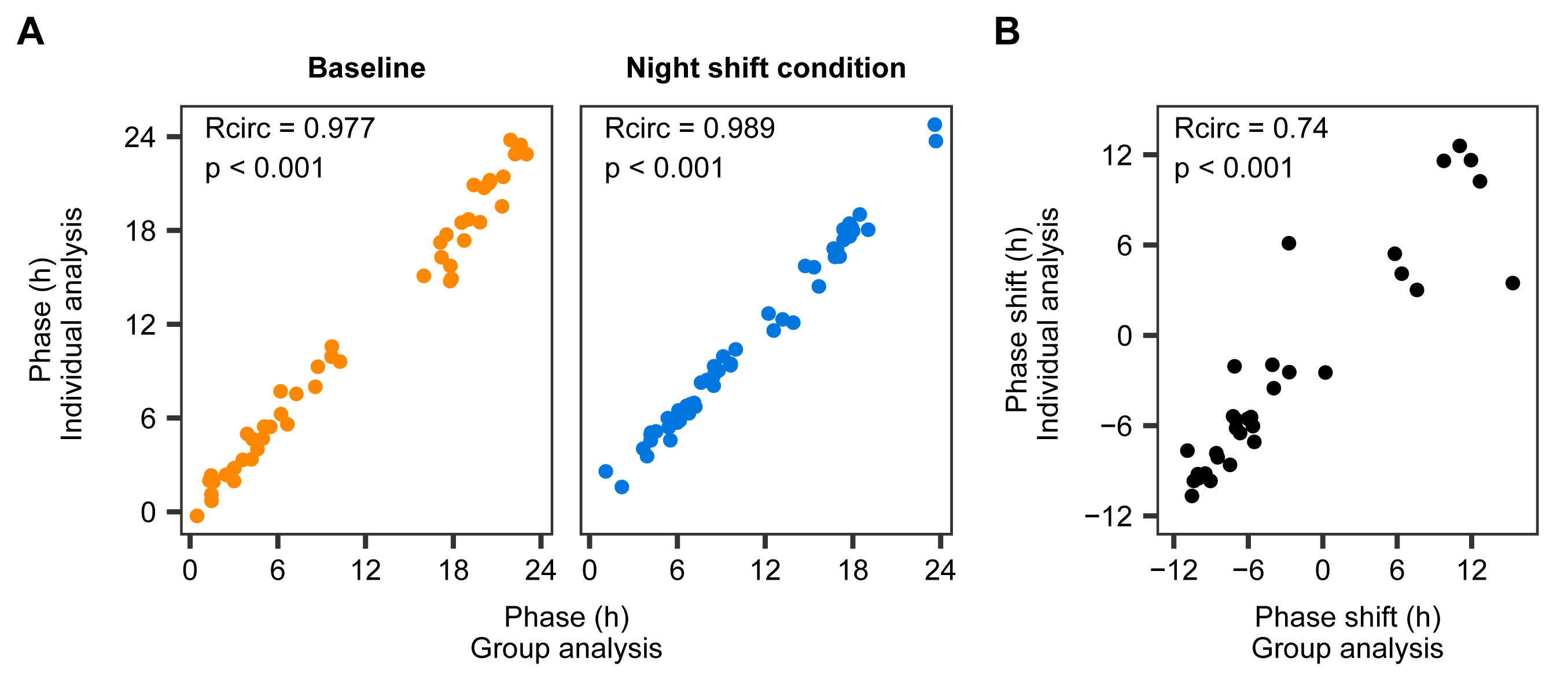

Supplement: S5 Fig — Coherence of phase estimates (A) and phase shifts (B) derived from the group and individual analyses. (A) Correlation between phase estimates derived from the group cosinor analysis and individual cosinor analysis of the metabolites identified as rhythmic at baseline (n = 51) and during the night shift condition (n = 53). The average phases of the individual analysis were obtained by computing the circular mean of the phase estimates of all individually rhythmic time series (uncorrected p-value <0.05) per metabolite per condition. (B) Correlation between the phase shifts derived from the group cosinor analysis and individual cosinor analysis of the 32 commonly rhythmic metabolites. Phase shifts on the group level were calculated by subtracting the phase during the night shift condition from the phase at baseline for each metabolite. Phase shifts on the individual level were obtained by computing the phase shift per subject per metabolite, after which the average phase shift (circular mean) across subjects was calculated. Only individually rhythmic time series (uncorrected p-value <0.05) were used to compute phase shifts per subject. Numerical data underlying the results presented in this figure are available in S2 Data. p, significance of the correlation coefficient; Rcirc, circular version of the Pearson product-moment correlation. (TIF) [file pbio.3000303.s005.tif]
