# Supplementary material for: Individual metabolomic signatures of circadian misalignment during simulated night shifts in humans
Source: PLoS Biol. 2019 Jun 18;17(6):e3000303. doi: 10.1371/journal.pbio.3000303 (PMC6581237; doi:10.1371/journal.pbio.3000303)
Supplement: S1 Table — (DOCX) [file pbio.3000303.s006.docx]

**Table S1 – Class overrepresentation analysis**

**Table S1A –** Class representation analysis of metabolites that are rhythmic at baseline but not during the night shift condition (n = 19)

| Metabolite class | Number of metabolites in class | Expected | Number of metabolites that lose rhythmicity | P-value (Fisher’s Exact test) |
| --- | --- | --- | --- | --- |
| Acylcarnitine | 40 | 5.846154 | 1 | 0.999524 |
| Amino acid | 24 | 3.507692 | 3 | 0.729668 |
| Biogenic Amine | 12 | 1.753846 | 2 | 0.551169 |
| Lysophospholipid | 14 | 2.046154 | 6 | 0.006346 |
| Organic Acid | 16 | 2.338462 | 2 | 0.720309 |
| Other | 4 | 0.584615 | 1 | 0.472747 |
| Phosphatidylcholine | 10 | 1.461538 | 3 | 0.16273 |
| Sphingolipid | 10 | 1.461538 | 1 | 0.806529 |

**Table S1B –** Class representation analysis of metabolites that are rhythmic at baseline but not during the night shift condition (n = 21).

| Metabolite class | Number of metabolites in class | Expected | Number of metabolites that gain rhythmicity | P-value (Fisher’s Exact test) |
| --- | --- | --- | --- | --- |
| Acylcarnitine | 40 | 6.461538 | 11 | 0.020881 |
| Amino acid | 24 | 3.876923 | 6 | 0.158637 |
| Biogenic Amine | 12 | 1.938462 | 1 | 0.891269 |
| Lysophospholipid | 14 | 2.261538 | 0 | 1 |
| Organic Acid | 16 | 2.584615 | 2 | 0.775422 |
| Other | 4 | 0.646154 | 0 | 1 |
| Phosphatidylcholine | 10 | 1.615385 | 1 | 0.839962 |
| Sphingolipid | 10 | 1.615385 | 0 | 1 |

**Table S1C -** Class representation analysis of metabolites with significantly increased levels during the night shift condition (n = 7)

| Metabolite class | Number of metabolites in class | Expected | Number of metabolites with increased levels | P-value (Fisher’s Exact test) |
| --- | --- | --- | --- | --- |
| Acylcarnitine | 40 | 2.153846 | 0 | 1 |
| Amino acid | 24 | 1.292308 | 1 | 0.769305 |
| Biogenic Amine | 12 | 0.646154 | 1 | 0.500913 |
| Lysophospholipid | 14 | 0.753846 | 4 | 0.002531 |
| Organic Acid | 16 | 0.861538 | 1 | 0.61049 |
| Other | 4 | 0.215385 | 0 | 1 |
| Phosphatidylcholine | 10 | 0.538462 | 0 | 1 |
| Sphingolipid | 10 | 0.538462 | 0 | 1 |

**Table S1D -** Class representation analysis of metabolites with significantly decreased levels during the night shift condition (n = 20)

| Metabolite class | Number of metabolites in class | Expected | Number of metabolites with decreased levels | P-value (Fisher’s Exact test) |
| --- | --- | --- | --- | --- |
| Acylcarnitine | 40 | 6.153846 | 6 | 0.626159 |
| Amino acid | 24 | 3.692308 | 3 | 0.765082 |
| Biogenic Amine | 12 | 1.846154 | 3 | 0.272905 |
| Lysophospholipid | 14 | 2.153846 | 0 | 1 |
| Organic Acid | 16 | 2.461538 | 4 | 0.213031 |
| Other | 4 | 0.615385 | 2 | 0.111745 |
| Phosphatidylcholine | 10 | 1.538462 | 0 | 1 |
| Sphingolipid | 10 | 1.538462 | 2 | 0.475362 |

**Table S1E -** Class representation analysis of metabolites identified as behavior-influenced (n = 24)

| Metabolite class | Number of metabolites in class | Expected | Number of behavior-influenced metabolites | P-value (Fisher’s Exact test) |
| --- | --- | --- | --- | --- |
| Acylcarnitine | 40 | 7.384615 | 8 | 0.469491 |
| Amino acid | 24 | 4.430769 | 9 | 0.012114 |
| Biogenic Amine | 12 | 2.215385 | 1 | 0.923636 |
| Lysophospholipid | 14 | 2.584615 | 1 | 0.951646 |
| Organic Acid | 16 | 2.953846 | 4 | 0.33644 |
| Other | 4 | 0.738462 | 1 | 0.56266 |
| Phosphatidylcholine | 10 | 1.846154 | 0 | 1 |
| Sphingolipid | 10 | 1.846154 | 0 | 1 |

**Table S1F -** Class representation analysis of metabolites identified as circadian-influenced (n = 7)

| Metabolite class | Number of metabolites in class | Expected | Number of circadian-influenced metabolites | P-value (Fisher’s Exact test) |
| --- | --- | --- | --- | --- |
| Acylcarnitine | 40 | 2.153846 | 0 | 1 |
| Amino acid | 24 | 1.292308 | 3 | 0.116963 |
| Biogenic Amine | 12 | 0.646154 | 1 | 0.500913 |
| Lysophospholipid | 14 | 0.753846 | 0 | 1 |
| Organic Acid | 16 | 0.861538 | 2 | 0.206554 |
| Other | 4 | 0.215385 | 1 | 0.200746 |
| Phosphatidylcholine | 10 | 0.538462 | 0 | 1 |
| Sphingolipid | 10 | 0.538462 | 0 | 1 |
